# A Density Functional Theory Study on the Deformation Behaviors of Fe-Si-B Metallic Glasses

**DOI:** 10.3390/ijms130810401

**Published:** 2012-08-21

**Authors:** Guang-Ping Zheng

**Affiliations:** Department of Mechanical Engineering, The Hong Kong Polytechnic University, Hung Hom, Kowloon, Hong Kong; E-Mail: mmzheng@polyu.edu.hk; Tel.: +852-27666660; Fax: +852-23654703

**Keywords:** density functional theory, molecular dynamics, metallic glasses, defects in solids

## Abstract

Density functional theory has been employed to investigate the deformation behaviors of glassy Fe-Si-B model systems prepared by *ab initio* molecular dynamics. The atomistic deformation defects which are closely related to the local dilation volumes or excess volumes and unstable bonding have been systematically analyzed. It has been found that the icosahedral structures are relatively stable under shear deformation until fracture occurs. Plastic flow is indicated by interruption of percolating icosahedral structures, caused by unstable Fe-Si bonding of *p*-*s* hybridization in nature.

## 1. Introduction

Metallic glasses are non-crystalline alloys with many attractive mechanical properties such as high fracture toughness, exceptionally high tensile strength, and outstanding super-plasticity below their glass transition temperatures [1]. Compared with conventional crystalline metallic materials, metallic glasses do not have dislocation defects during the deformation process and the deformation is localized, which results in very little plasticity at room temperature. Such deformation behaviors have prevented metallic glasses from wide-spread structural applications. Without atomistic details on deformation mechanisms of metallic glasses from experiments and simulation investigations, it is really difficult to develop metallic glasses with combined excellent mechanical properties especially improving their tensile ductility. Thus, significant efforts have been made to understand the local order/disorder atomic structures of metallic glasses [2] and the deformation defects or plastic flow defects which are responsible for their plastic deformation, usually in the form of shear banding. Unfortunately, unlike crystalline materials, metallic glasses possess no static dislocation-like flow defects per se which can be imaged via electron microscopy. Density functional theory (DFT) combined with the pseudopotential method has been adopted to investigate the atomistic details of the local short-range and medium-range orders in metallic glasses [3,4], and could be used to understand their deformation defects, since the only inputs are the atom species, atomic coordinates and number. It gives a reliable and accurate description of the atomic structures, energetics and electronic structures of the modeled materials under mechanical deformation.

It is convenient to define the loosely packed atomic clusters that are responsible for the plastic deformation as deformation defects in metallic glasses. In fact, the concepts of deformation defects have been proposed and developed by Spaepen as the free-volume zones [5] and by Argon as shear transformation zones [6]. In the former model, the size of the defective region where there are excess volumes or dilatation volumes for the atoms to move under local shear strain is typically below 0.3 nm. Density functional theory could provide deep insight into the chemical and structural defects that are vulnerable to mechanical deformation rather than the ideal glassy structures usually defined as icosahedral clusters, although the size of the system that can be handled is smaller than several nanometers.

The iron-based metallic glasses are superior to their metallic glass counterparts because theoretically they possess greater strength and because their main content iron is relatively cheap [7]. However understanding the brittle nature of these amorphous alloys at the atomistic level is still lacking, which hinders the design of iron-based metallic glasses with excellent combined mechanical strength and ductility. In this work, we investigate the deformation behaviors of an experimentally well-investigated iron-based amorphous Fe-Si-B alloy system using *ab initio* molecular dynamics which is primarily based on DFT. Several types of stable glassy systems with different characteristic excess volumes or dilatation volumes, *i.e.*, glassy alloys under ambient pressure and different hydrostatic pressures, were prepared for shear deformation. The responses of the free-volume zones and the ideal glassy structure in these systems under shear deformation were determined. It was thought such investigations could determine the relation between mechanical properties of metallic glass and its deformation defects from first principles.

## 2. Simulation Methods

Fe_80_Si_11_B_9_ supercell consisting of 200 atoms was chosen for the simulation which was performed using first-principles simulation code VASP [8]. Generalized gradient approximation for the exchange-correlation energy and the spin polarized projector augmented-wave method was implemented. Kohn-Sham single electron wave functions were expanded by plane waves with well-converged cut-off energy of 400 eV. The Brillouin zone sampling consists of the k-point only. The 2 × 2 × 2 k-point mesh was used to calculate the densities of states of the systems. The metallic glass was prepared by quenching the melt to room temperature. The starting configuration of the Fe-Si-B system for simulation was generated by classical molecular dynamics (MD) simulation of Fe-Si-B alloys using hard sphere potentials. In *ab initio* MD simulation the system was first melted at *T* = 1500 K for 5 ps and is then quenched to *T* = 650 K with a cooling rate of 2 × 10^14^ K/s. The system with a density of 7.02 kg/m^3^ was maintained as undercooled liquid for 1.5 ps, followed by quenching to *T* = 300 K with a quenching rate of 4 × 10^14^ K/s. All the *ab initio* MD simulations were performed in the canonical (NVT) ensemble and a Nose thermostat was used for temperature control. A stable glassy Fe_80_Si_11_B_9_ system was finally obtained by relaxations of atomic coordinates and supercell volume using the conjugate gradient algorithm at *T* = 0 K and at a hydrostatic pressure of *P* = 100 kPa. Fe_80_Si_11_B_9_ glassy alloys with different densities which are subjected to hydrostatic pressures of *P* = 22.02 GPa, −13.13 GPa and −19.50 GPa were prepared using the same procedures. The tolerances of 1.0 meV for maximal change in total energy and 10 MPa for maximal change in stress components were used to determine a stable glassy system.

Deformation of the glassy alloys whose dimensions are about 1 × 1 × 2 nm^3^ was performed by applying shear strain γ_xz_ at *T* = 0 K. Atoms are relaxed in the micro-canonical (NVE) ensemble until the fluctuations of the total energy and stress components of the system are within the tolerances.

## 3. Results and discussions

**Figure 1:**
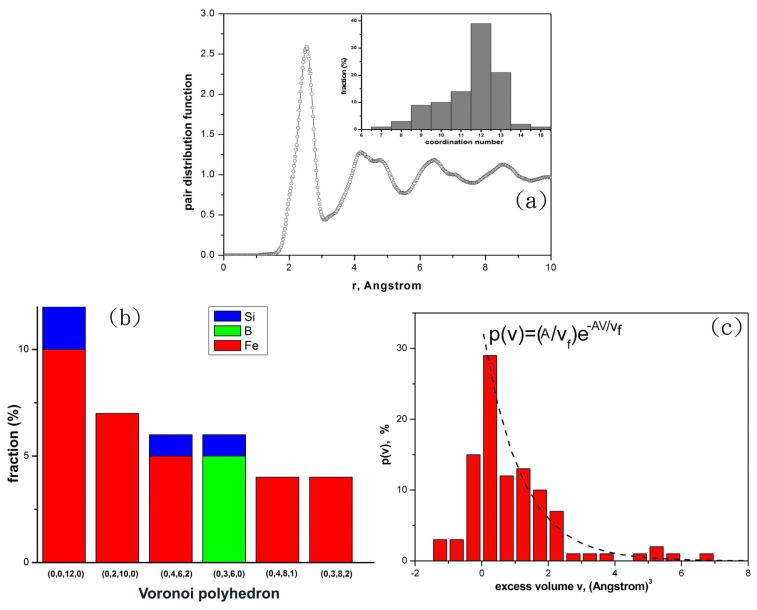
Analyses on the structural properties of Fe-Si-B glassy alloy at ambient pressure. (**a**) Pair distribution function. The inset is the histogram of atomic coordination numbers. (**b**) Fraction of Voronoi polyhedrons. (**c**) Histogram of excess volumes *v*. The dash curves are the fits for histogram of *v* > 0.

Figure 1a shows the pair distribution function of the Fe_80_Si_11_B_9_ systems at ambient pressure (*P* = 100 kPa) and at *T* = 0 K, suggesting the alloy systems are fully amorphous. Voronoi tessellation can be used to extract local geometric structures among neighboring atoms [9]. The Voronoi cell or Voronoi polyhedron of an atom can be labeled as indices (I_3_, I_4_, I_5_, I_6_) where I_i_ is the number of faces of *i* edges in the Voronoi polyhedron. It has been revealed that the Voronoi icosahedron indexed as (0, 0, 12, 0) has the prevailing short range order (SRO) in metallic glasses, and it is generally considered as a unit cell of an ideal glassy system [9]. Moreover the volume of the Voronoi cell of an atom has been applied to quantitatively measure the local excess volume or dilatation volume of such an atom [10]. To analyze how the excess volumes of atoms change during the mechanical deformation process, one could define the local excess volume of an atom as the volume of its Voronoi cell subtracted from the averaged volume of Voronoi icosahedrons of the same species in the stable and un-deformed glassy alloy. In this work, we define the averaged volume of Voronoi icosahedrons as the average of volumes of all Voronoi icosahedrons.

The histogram plots of the coordination numbers of atoms are shown in the inset of Figure 1a. Figure 1b shows the fractions of several types of Voronoi polyhedrons in the glass alloy. Here the most stable glassy alloy obtained from the *ab initio* MD simulation is Fe_80_Si_11_B_9_ under ambient pressure since its free energy is the smallest compared with those of Fe_80_Si_11_B_9_ glassy alloys under different hydrostatic pressures. Figure 1c shows the histogram of the excess volumes *v* of atoms which are defined as the volumes of Voronoi cells subtracted from the average volume of the icosahedrons. For those Voronoi polyhedrons with their volumes larger than the average volume of the icosahedrons (*v* > 0), the histogram can be fitted with an exponential probability function *P*(*v*) = (*A*/*v*_f_)exp(−*Av*/*v*_f_) with the fitting parameters *A* and *v*_f_. The fitting parameter *v*_f_ = 1.15 × 10^−3^ nm^3^ can be defined as the average excess volume that an atom can be squeezed into during the mechanical deformation process.

**Figure 2:**
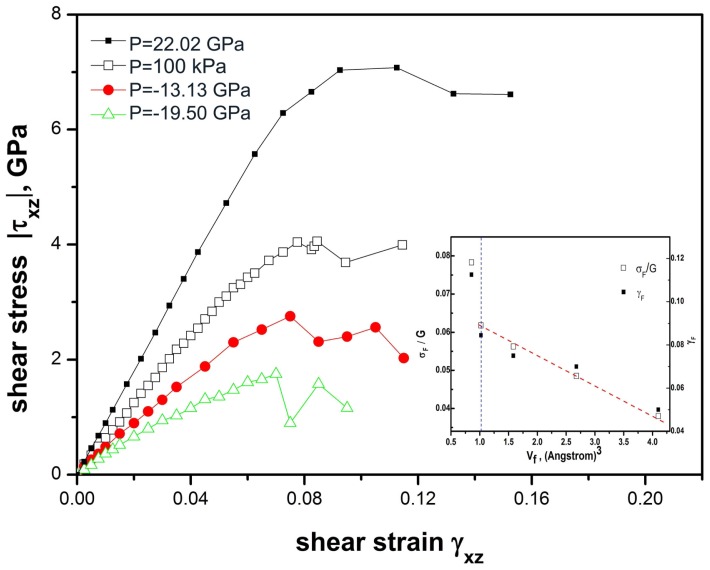
Stress-strain relation of the deformed Fe-Si-B glassy alloys at different hydrostatic pressures *P*. The inset shows the dependences of shear strength *σ*_F_/*G* and flow strain *γ*_F_ on the average excess volumes v_f_ of the glassy alloys. The blue dashed line indicates the threshold above which *σ*_F_/*G* and *γ*_F_ depend linearly on *v*_f_.

The stress-strain relations of Fe_80_Si_11_B_9_ metallic glasses at different hydrostatic pressures are shown in Figure 2. It can be found from the stress-strain relation of the deformed glassy system at ambient pressure that the flow stress is *σ*_F_ = 4.06 GPa at a shear strain *γ*_F_ = 0.0845, beyond which the system is unstable toward fracture at a fracture strain of 0.12. The shear modulus is given by *G* = 62.47 GPa which is comparable with the experimental one [11]. The large plasticity, however, as seen in the simulated deformation processes is different from the experiments. This could be mainly caused by finite size effects on the deformation defects in the simulation and deserves further investigation. Fe_80_Si_11_B_9_ glassy alloy subjected to hydrostatic pressure of *P* = 22.02 GPa shows higher shear modulus, flow strain and flow stress than those of the system at ambient pressure. While these three important mechanical properties of the glassy alloys subjected to hydrostatic pressures of *P* = −13.13 GPa and −19.50 GPa are significantly lower than those of the system at ambient pressure. The dependences of *σ*_F_/*G* and *γ*_F_ on the average excess volumes *v*_f_ of the glassy alloys are shown in the inset of Figure 2. Both the shear strength *σ*_F_/*G* and the flow strain *γ*_F_ increase linearly with decreasing *v*_f_, suggesting that *v*_f_ could be used as a meaningful measure of the atomistic deformation defect whose size determines the mechanical properties of the glassy alloy.

**Figure 3:**
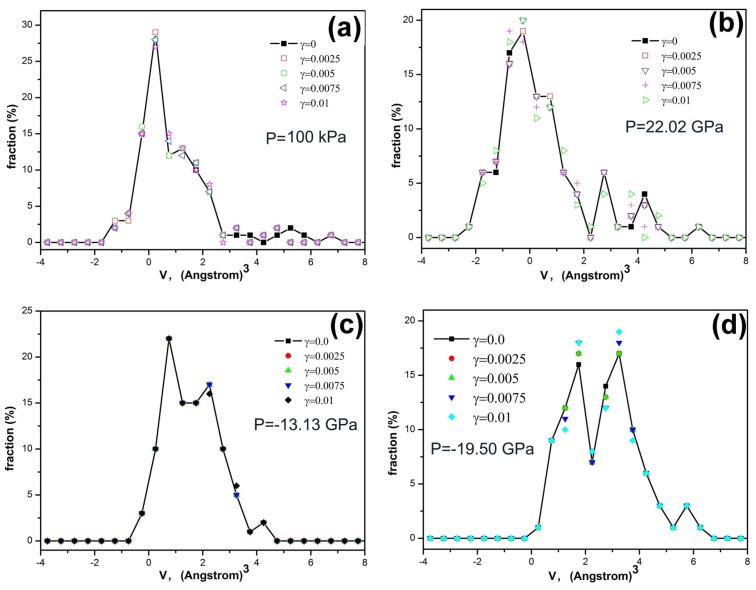
(**a**–**d**) Histograms of excess volumes *v* in Fe-Si-B glassy alloys at different hydrostatic pressures *P* and under various elastic shear strains.

**Figure 4:**
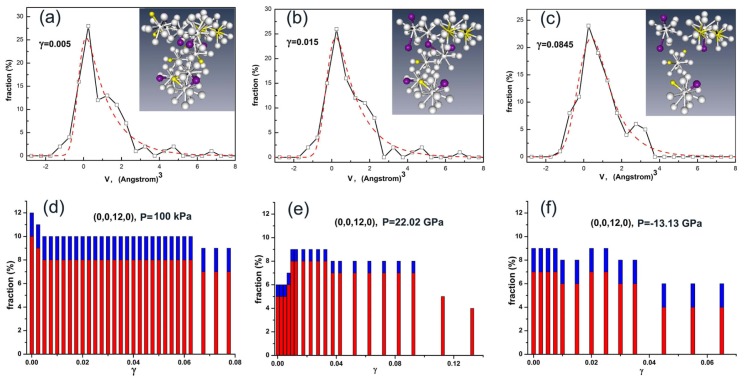
(**a**–**c**) Histograms of excess volumes *v* in Fe-Si-B glassy alloy at ambient pressure under shear strains *γ* = 0.005, 0.015 and 0.0845, respectively. The dashed red curves are fits using a modified exponential function. The insets are icosahedral clusters of atoms with grey, yellow and brown spheres representing Fe, B and Si atoms, respectively. (**d**–**f**) The effect of shear strain on the number of fractions of Voronoi icosahedrons in Fe-Si-B glassy alloys at different hydrostatic pressures P.

The effect of excess volume *v* on the mechanical properties can be elucidated by the analyses on the histogram distributions of excess volumes in different glassy alloys under elastic shear strains, as shown in Figure 3. The distributions of excess volumes in Fe_80_Si_11_B_9_ metallic glass subjected to shear strains of *γ* = 0–0.01 at ambient pressure are shown in Figure 3a. Compared with a modified exponential fitting function as shown as the red dash curve in Figure 4a, it can be seen that there is an inherent non-zero excess volume of *v*_f_ = 1.25 × 10^−3^ nm^3^, manifesting itself by a peak in the distribution function, which does not change with the applied elastic strains, but such a characteristic peak or the value of excess volume changes with applied plastic strains as shown in Figure 4a–c. These results demonstrate that atomic sites with excess volumes close to *v*_f_ = 1.25 × 10^−3^ nm^3^ are vulnerable to plastic deformation. Hence it is reasonable to consider those atomic sites as deformation defects which lead to plastic flow. For Fe_80_Si_11_B_9_ glassy alloy at a hydrostatic pressure of *P* = 22.02 GPa, there is no obvious peak in the distribution function of excess volumes, as shown in Figure 3b. For Fe_80_Si_11_B_9_ glassy alloy at hydrostatic pressures of *P* = −13.13 GPa and −19.50 GPa, there is more than one peak in their distribution functions of excess volumes, as shown in Figure 3c,d, respectively. The results further demonstrate that some atomic sites should exist, vulnerable to plastic deformation which could be considered as deformation defects. Fe_80_Si_11_B_9_ glassy alloys subjected to various hydrostatic pressures show a decrease in the shear strength *σ*_F_/*G* and the flow strain *γ*_F_ when they have more vulnerable sites or deformation defects. This provides an atomistic explanation of the mechanical properties of the glassy alloys as shown in the inset of Figure 2.

Although it is difficult to determine the details of the atomic structure of the deformation defects defined from the above analyses, basically the deformation defects should not be the atomic sites that possess Voronoi icosahedrons or zero excess volume. This could be demonstrated in Figure 4d–f which shows the number of Voronoi icosahedrons under shear strains in the glassy alloys. It was found that the structures of icosahedral clusters of atoms (atoms at the vertexes of Voronoi icosahedrons) are relatively stable under shear deformation. The number of Voronoi icosahedrons significantly changes only when fracture occurs, as shown in Figure 4d–f. The evolutions of the icosahedral clusters of atoms under shear deformation are shown in the insets of Figure 4a–c. It was found that the icosahedral clusters of atoms form a percolating cluster across the glassy system as shown in the inset of Figure 4a–b, which is ruptured at fracture as shown in the inset of Figure 4c.

**Figure 5:**
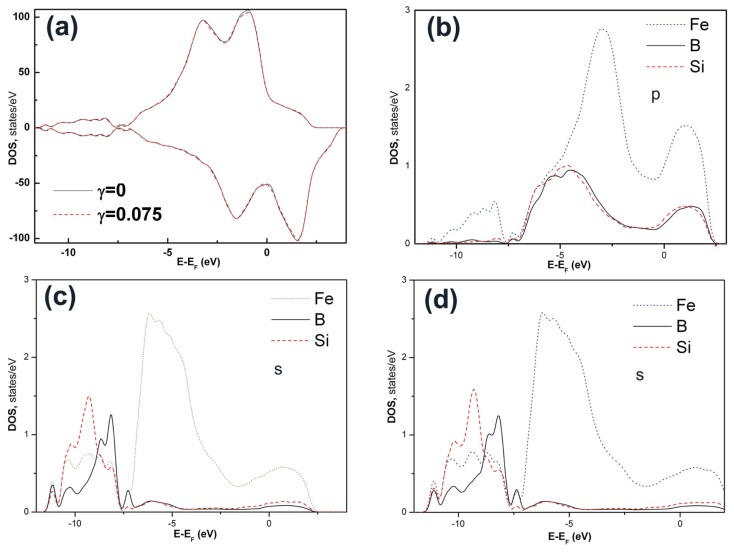
(**a**) Total density of states (DOS) of Fe-Si-B un-deformed and deformed (shear strain 0.075) glassy alloy at ambient pressure. The DOS is positive for majority-spin states and negative for minority-spin states. (**b**,**c**) Local DOS of *p* electrons and *s* electrons of the atoms in the un-deformed Fe-Si-B glassy alloy, respectively. (**d**) Local DOS of *s* electrons of atoms in the deformed (shear strain 0.075) Fe-Si-B glassy alloy.

**Figure 6:**
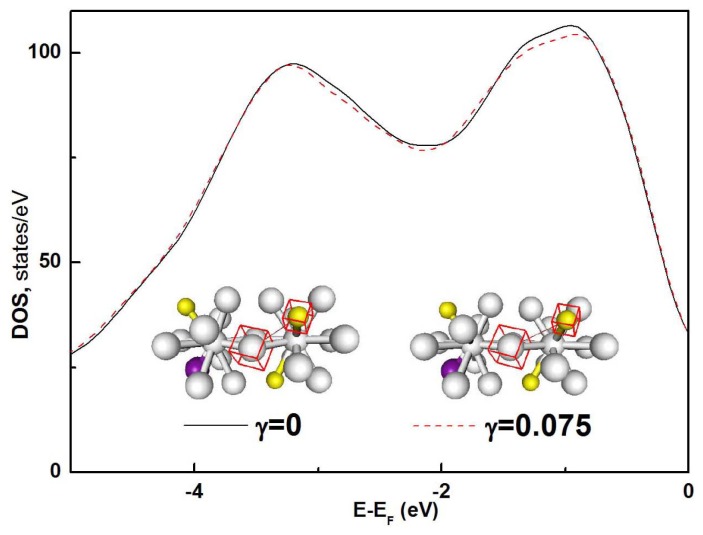
The majority-spin DOS of un-deformed (*γ* = 0) and deformed (*γ* = 0.075) Fe-Si-B glassy alloys at 0.4–3.0 eV below the Fermi level. The insets show the icosahedral clusters of atoms in the glassy alloys. Grey, yellow and brown spheres represent Fe, B and Si atoms, respectively. The Fe-B bond whose bond length changes with applied shear strain is indicated by a thin line and atoms of this bond are indicated by the red boxes.

Besides the structural and volumetric details of the deformation defects, it is important to see how the electronic structure of the glassy alloy responds to mechanical deformation. Figure 5a shows the total density of states (DOS) of Fe_80_Si_11_B_9_ glassy alloy at ambient pressure under a shear strain of *γ* = 0.075. Compared with that of the un-deformed system (γ = 0), the majority-spin DOS of the system under *γ* = 0.075 reduces at 0.4–3.0 eV below the Fermi level E_F_. The close view on such reduction is shown in Figure 6. The local densities of states (LDOS) of the deformed and un-deformed systems are shown in Figure 5b–d. The peaks of LDOS at −11 eV suggest that the Fe-Si bonding is *p*-*s* hybridized and is unstable against large shear strains.

The reduction of the majority-spin total DOS of the system under a shear strain of *γ* = 0.075 at *E*-*E*_F_ = 0.4–3.0 eV might be related to the changes of *T*_2g_ and *E*_g_ symmetry states in body center cubic Fe, since the electrons of the Fe atoms bonding with other atoms contribute mostly to the total DOS in this energy range. The changes could be caused by the significant elongation of Fe-B bonds in the icosahedron clusters under shear strain, as shown in the insets of Figure 6. The distance between Fe and B atoms (indicated by red boxes in the insets of Figure 6) is significantly increased from 0.275 nm to 0.31 nm when the applied shear is larger than the flow stress *σ*_F_, while other bonds remain almost unchanged. The icosahedrons are forced apart if the Fe-B bond length is larger than 0.3 nm.

## 4. Conclusions

Density functional theory has been employed to investigate the deformation behaviors of glassy Fe-Si-B model systems. The atomic sites that are vulnerable to plastic deformation show a common excess volume, and are considered as flow defects in glassy alloys. Using the excess volume of atomic sites as a measure to characterize the deformation defects, the mechanical properties of glassy alloys subjected to various hydrostatic pressures were found to be successfully connected to the number of deformation defects and their characteristic volume. It was found that the structures of icosahedral clusters of atoms are relatively stable under shear deformation until fracture occurs. Plastic flow is indicated by interruption of percolating icosahedral clusters of atoms, caused by unstable Fe-Si bonding of *p*-*s* hybridization in nature. The breaking of Fe-B bonds in the percolating icosahedral clusters of atoms could result in fractures of glassy alloys. The density functional theory thus describes accurately the details of the atomic structures, energetics and electronic structures of the modeled metallic glasses under mechanical deformation, which cannot be provided by experiments and other simulation methods.
